# Adaptive Evolution of Odorant-Binding and Chemosensory Protein Gene Families in Genus *Drosophila* Fallén, 1823 (Diptera, Drosophilidae)

**DOI:** 10.3390/biom16020330

**Published:** 2026-02-20

**Authors:** Xing-Yu Pang, Si-Yang Liu, Quan-You Yu, Shou-Min Fang

**Affiliations:** 1 Key Laboratory of Southwest China Wildlife Resources Conservation (Ministry of Education), College of Life Science, China West Normal University, Nanchong 637002, China; 13953166102@163.com; 2 School of Life Sciences, Chongqing University, Chongqing 400044, China; 20205578@stu.cqu.edu.cn

**Keywords:** *Drosophila*, odorant-binding protein, positive selection, gene expansion, molecular docking

## Abstract

Odorant-binding proteins (OBPs) and chemosensory proteins (CSPs) serve as carriers for signal molecules within the insect olfactory system, playing a crucial role in detecting chemical cues related to feeding and reproduction. However, their roles in host shift and environmental adaptation remain poorly understood. This study identified the OBP and CSP gene families across 32 *Drosophila* species, revealing their adaptive evolutionary trajectory. It was found that the gene number of the OBP family varied widely between species, ranging from 37 to 66 genes, while the gene number of the CSP family was conserved. The OBP family experienced two major gene expansion events on the ancestral branches of the diet-diverse *melanogaster* lineage, leading to an increased number. Positive selection occurred during evolution in the orthologous groups of *Obp22a*, *Obp57e* and *Obp83ef*. Homology modeling and molecular docking revealed that variation in the positively selected sites across different *Drosophila* species resulted in significant changes to free binding energy and affinity for plant odors and insecticides. Our findings highlight gene expansion and functional diversification within the *Drosophila* OBP family may contribute to shaping the dietary spectrum and promoting adaptation to toxic substances.

## 1. Introduction

Insect olfaction is crucial for detecting and perceiving environmental odors, which plays an important role in food localization, reproductive behavior, and avoiding harmful smells [1,2]. Odor recognition and behavioral response are mediated by highly complex signal transduction processes that primarily occur in the peripheral antennae and the central nervous system [2,3]. The olfactory sensilla at the peripheral antennae serve as the fundamental units, which mediate the process of odor recognition. In general, pheromones and host plant volatiles enter the sensillar lymph, where they are transported to olfactory receptor neurons (ORNs) by odorant-binding proteins (OBPs) and chemosensory proteins (CSPs) [3]. Then, the ORs are activated by odor molecules, generating odor-evoked potentials [3]. Therefore, OBPs and CSPs play a crucial role in the peripheral olfactory perception in insects, which is essential for mating, feeding, and oviposition [4,5].

In addition to carrying odor molecules, OBPs and CSPs may also participate in other physiological processes within the olfactory system and other tissues [6,7,8,9,10]. Research has found that toxic substances can enter peripheral olfactory organs. OBPs and CSPs competitively bind to toxic molecules in sensilla, including insecticides, thereby maintaining olfactory sensitivity [6,7]. For instance, multiple insecticides have been shown to induce OBP and CSP gene expression in *Spodoptera litura* (Fabricius, 1775) (Lepidoptera, Noctuidae), and RNA interference (RNAi) knockdown significantly reduces survival rates [11]. Except for the olfactory organs, OBP and CSP genes are also expressed in other tissues, suggesting that they may participate in other physiological functions [12,13]. For example, *Drosophila Obp56g* shows high expression in the male reproductive tract, which is involved in the formation of mating plug, ejaculate retention, and sperm storage [8]. OBP genes have also been associated with gustatory responses to tastants [14] and lifespan [12]. It has been postulated that CSPs expressed in non-olfactory tissues are involved in carbon dioxide detection, larval development and leg regeneration [13].

*Drosophila melanogaster* (Meigen, 1830) (Diptera, Drosophilidae) is a well-studied model insect. Research on its OBPs and CSPs has revealed insights into the molecular transport functions of odorant molecules [14,15,16,17,18]. Fifty-two OBP genes were identified in the *D. melanogaster* genome [17,19], mainly classified into Classic, Minus-C, ABPII, CRLBP, Dimer, and Plus-C subfamilies [20]. Obp76a and Obp69a play a crucial role in the electrophysiological activation of chemosensory neurons and in the response to the male-specific pheromone cis-vaccenyl acetate (cVA) during mating and aggression behavior [21,22]. Additionally, Obp56h may bind to the male-produced inhibitory pheromone 5-triene (5-T) [23]. RNAi knocking down *Obp56h* expression enhances mating behavior by reducing courtship latency [23]. Obp57d and Obp57e play crucial roles in detecting hexanoic and octanoic acids. These acids are involved in differentiating oviposition site preferences between *D. melanogaster* and *D. sechellia* [24]. Obp28a is involved in detecting the floral odour ß-ionone [25]. Compared to OBPs, CSP family contains only four genes in the *D. melanogaster* genome, and their functional validation remains limited [18].

Research on the phylogeny, feeding habits, and geographic distribution of the genus *Drosophila* Fallén, 1823 has spanned over a century [26]. The genus *Drosophila* belongs to the subfamily Drosophilinae and comprises approximately 2000 species. *Drosophila* species primarily cluster into *melanogaster*, *repleta*, *virilis*, *obscura*, *immigrans*, *quinarian*, and *testacea* groups [26,27,28]. Their geographic distributions vary considerably, leading to variations in food sources [27]. For example, the *melanogaster* group is primarily distributed in Australasia, Indomalaya, and Africa, and its food sources mainly include fruits, flowers, cacti, and other plant parts [29]. The *repleta* group is one of the major species radiations in the genus *Drosophila* and is highly adapted to a cactophilic lifestyle; that is, its members use necrotic cacti as breeding, mating, and feeding sites [29]. cVA, the most extensively studied component of the aggregation pheromone, is primarily found in the *melanogaster*, *immigrans*, *quinarian*, and *testacea* groups [28]. In other species, the pheromone compounds that promote aggregation are typically volatile esters, ketones, or unsaturated hydrocarbons [28,30,31]. Host shifts and differences in pheromone components will inevitably lead to adaptive differentiation in olfactory perception [32]. OBPs and CSPs, as key components involved in odor transport, may undergo gene expansion and/or natural selection during *Drosophila* speciation and adaptation [12,19,33,34].

As whole-genome sequencing becomes more cost-effective, more and more *Drosophila* species are being sequenced. This paves the way for exploring the molecular evolution of OBPs and CSPs in ecological, adaptive, and behavioral differentiation among *Drosophila* species. This study identified the OBP and CSP gene families across 32 *Drosophila* species at the genome-wide level. Through analyses of gene gain and loss, as well as evolutionary rates, the study revealed the evolutionary impact of habitat and host use on OBPs and CSPs. In particular, the positive selection of orthologous genes was detected, and molecular docking was used to evaluate the effect of positively selected sites on binding to plant volatiles and exogenous toxic insecticides. This study enhances our understanding of the importance of gene expansion and functional diversification within the OBP and CSP families in shaping the dietary spectrum and promoting adaptation to toxic substances.

## 2. Materials and Methods

### 2.1. Genomic Data

**Figure 1:**
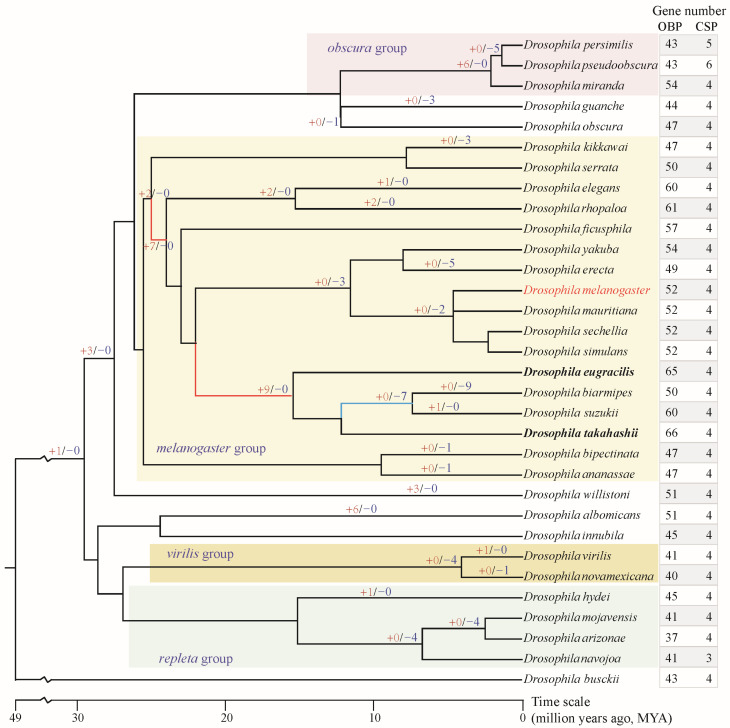
Gene number of OBP and CSP families in genus *Drosophila* and gene gain/loss of OBPs. The species tree was obtained from the TimeTree database (http://www.timetree.org/ (accessed on 1 October 2025)). The “+red number” on the branch indicates the number of OBP genes gained, while the “−blue number” indicates the number of OBP genes lost. Unlabeled branches indicate no gene gain or loss occurred. The red branches in the *melanogaster* group indicate greater expansion of the OBP gene; the blue branch indicated greater loss of the OBP gene. The bold species indicate the highest total number of OBP genes.

In this study, 32 representative species were selected from genus *Drosophila*, including *D. melanogaster* (Meigen, 1830), *D. albomicans* (Duda, 1923), *D. ananassae* (Doleschall, 1858), *D. arizonae* (Ruiz, Heed & Wasserman, 1990), *D. biarmipes* (Malloch, 1924), *D. bipectinata* (Duda, 1923), *D. busckii* (Coquillett, 1901), *D. elegans* (Bock & Wheeler, 1972), *D. erecta* (Tsacas & Lachaise, 1974), *D. eugracilis* (Bock & Wheeler, 1972), *D. ficusphila* (Kikkawa & Peng, 1938), *D. guanche* (Monclus, 1977), *D. hydei* (Sturtevant, 1921), *D. innubila* (Spencer, 1943), *D. kikkawai* (Burla, 1954), *D. mauritiana* (Tsacas & David, 1974), *D. miranda* (Dobzhansky, 1935), *D. mojavensis* (Patterson, 1940), *D. navojoa* (Ruiz, Heed & Wasserman, 1990), *D. novamexicana* (Patterson, 1941), *D. obscura* (Fallen, 1823), *D. persimilis* (Dobzhansky & Epling, 1944), *D. pseudoobscura* (Frolova, 1929), *D. rhopaloa* (Bock & Wheeler, 1972), *D. sechellia* (Tsacas and Bachli, 1981), *D. serrata* (Malloch, 1927), *D. simulans* (Sturtevant, 1919), *D. suzukii* (Matsumura, 1931), *D. takahashii* (Sturtevant, 1927), *D. virilis* (Sturtevant, 1916), *D. yakuba* (David, Debat & Yassin, 2017), *D. willistoni* (Sturtevant, 1916) (Figure 1). The annotated genes and proteins were retrieved from GenBank (https://www.ncbi.nlm.nih.gov/ (accessed on 2 March 2025)). Genome assembly information and download path were detailed in Table S1.

### 2.2. Identification of OBP and CSP Genes in Drosophila

We identified putative OBP and CSP members through similarity-based searches using BLAST v. 2.2.9 [35] and HMMER v3.3 (http://hmmer.wustl.edu/ (accessed on 1 June 2025)). First, we searched the predicted gene set with BLASTP (*e*-value threshold of 10^−5^) using the known OBPs and CSPs [36,37] as queries, as well as with HMMsearch (*e*-value threshold of 10^−5^) using HMM models of OBPs (PF01395) and CSPs (PF03392). In the second round, we used the identified OBPs and CSPs as queries to perform BLASTP searches (*e*-value threshold of 10^−5^) against the predicted gene sets of each *Drosophila* species. Due to the high diversity among OBP family members, we also constructed four extra HMM models for the known OBP sequences [36,37]. The known OBP sequences were clustered using BlastClust, with an *e*-value threshold of 10^−5^, length coverage “-L” of 0.5, and score density “-S” of 0.6. Four clusters with the highest numbers of sequences were selected. Each cluster was aligned separately with MAFFT [38] and constructed an HMM model using HMMER. HMMsearch was conducted again with an *e*-value threshold of 10^−5^. Finally, all the identified candidate OBPs were manually checked by performing HMMscan against the Pfam database v.33.1 [39] and Batch CD-Search against Conserved Domains Database (CDD) v.3.21 [40].

### 2.3. Reconstruction of Phylogenetic Tree and Gene Naming

Previous studies have systematically named the OBP and CSP families in *D. melanogaster* [36,37]. Using MAFFT software [38], the sequences of the named *D. melanogaster* OBPs and CSPs were aligned together with those of 31 other species, respectively. The alignments were trimmed by trimAl v1.4.1 (https://vicfero.github.io/trimal/ (accessed on 10 April 2025)) with gap threshold of 0.7. The maximum-likelihood (ML) phylogenetic trees were reconstructed by IQ-TRee v.1.6.12 with the optimal substitution model LG+I+G that provided the lowest Bayesian information criterion. In this study, we named each OBP and CSP gene according to its evolutionary relationship with *D. melanogaster* genes as shown in the phylogenetic trees.

### 2.4. Gene Gain and Loss Analysis of OBPs

ML tree of all the OBPs from 32 *Drosophila* species were reconstructed by IQ-TRee v.1.6.12. The species tree was obtained from the TimeTree database (http://www.timetree.org/ (accessed on 1 October 2025)). Gene gain and loss analysis was reconciled by Notung v2.9 software (http://www.cs.cmu.edu/~durand/Lab/Notung/ (accessed on 1 October 2025)) with default parameters.

### 2.5. Evolutionary Rate and Detection of Positive Selection

In this study, we identified 52 OBP and 4 CSP orthologous groups (OGs) based on the gene nomenclature and phylogenetic trees of all OBPs and CSPs. Evolutionary rates and positive selection detection were referred to in our previous study [41]. Briefly, nucleotide sequences were aligned using PAL2NAL (https://www.bork.embl.de/pal2nal/ (accessed on 10 April 2025)) to construct a multiple codon alignment from the corresponding aligned protein sequences of each OG. Synonymous (*d_N_*) and nonsynonymous (*d_S_*) substitution rates were estimated using the YN00 program implemented in the PAML 4.5 package [42], which simultaneously yielding the ω values (ω = *d_N_*/*d_S_*). The site model was used to detect signals of positive selection via likelihood-based model comparisons within the CODEML program of the PAML 4.5 package. Based on the posterior probabilities calculated using the BEB (Bayesian Empirical Bayes), sites with BEB ≥ 0.95 were considered to be under positive selection. Sites with BEB > 0.8 were regarded as candidate sites that are potentially under positive selection.

### 2.6. Homology Modeling and Molecular Docking

#### 2.6.1. Homology Modeling

The DmelObp22a, DmelObp57e and DmelObp83ef protein sequences were used to search for homology templates in SWISS-MODEL (http://swissmodel.expasy.org/interactive (accessed on 10 November 2025)). The results indicate that the optimal templates for these three OBP sequences are Q8MVX6.1.A, Q9V938.1.A, and Q9VNL2.1.A, respectively. The models were then downloaded and generated with SWISS-PDBVIEWER v4.1. The quality evaluation of the model structures was assessed by the Ramachandran plot generated by the PROCHECK module in SAVES v6.1 (https://saves.mbi.ucla.edu/ (accessed on 10 November 2025)).

#### 2.6.2. Molecular Docking

Plant-derived odor molecules and commonly used insecticides were used for the candidate ligands. Three-dimensional structures of ligands were downloaded from the PubChem database (https://pubchem.ncbi.nlm.nih.gov/ (accessed on 10 November 2025)). Molecular docking (MD) was referenced in the previous literature [43]. MD was performed using AutoDock v4.2 and AutoDock Tools v1.5.6. Water molecules were removed and polar hydrogen atoms were added, as well as the computed Gasteiger charge was added. The other parameters were set to their default values. The docking results were analyzed based on free binding energy; the conformation with the lowest binding energy was considered the most favorable. The 3D visualization of the docking results was performed using PyMOL v2.3 [43]. The 2D docking results was performed by Discovery Studio, using the CHARMm force field.

#### 2.6.3. Computational Site-Directed Mutagenesis

Computational site-directed mutagenesis was conducted to understand the effect of positive selection sites on binding activity. Combining computational alanine scanning (CAS) with MD methods determined the specific binding contributions of the positively selected sites with all ligands. Then, each positively selected site was individually mutated to alanine, and CAS was used to calculate the change in free energy (ΔG) before and after the mutation [44]. Subsequently, each positive selection site was mutated individually to variant amino acids found in other species. The ΔG before and after the mutation was calculated. Generally, a post-mutation energy value > 0.5 kcal/mol indicates an unstable structure and that the specific amino acid residue plays a crucial role in stabilizing the structure [44,45]. Conversely, a post-mutation energy value < −0.5 kcal/mol signifies that the structure remains stable and that the amino acid residue does not contribute to structural stability. Similarly, when the substitution energy change falls between 0.5 and −0.5 kcal/mol, it indicates that the amino acid has no significant effect on structural stability before and after mutation; that is, these amino acids play a minor role in the structural stability.

### 2.7. Temporal-Spatial Expression Profiles of CSP and OBP Genes in D. melanogaster

Flyatlas2 (https://motif.mvls.gla.ac.uk/FlyAtlas2/ (accessed on 5 November 2025)) contains various transcriptomic datasets for *D. melanogaster* across all developmental stages and tissues. We downloaded the FPKM (Fragments Per Kilobase of exon model per Million mapped fragments) values for each OBP and CSP gene. The expression heatmap was generated by Pheatmap package in R v4.3.1.

## 3. Results

### 3.1. Characterization of OBPs and CSPs Among Drosophila Species

Through comprehensive and manually curated searches, OBP and CSP genes were identified across the 32 analyzed *Drosophila* species (Tables S2 and S3, Figure 1), thereby improving the currently published data. Consistent with previous studies, *D. melanogaster* comprises 52 OBP and 4 CSP members [17,19], and no additional novel members were identified. Overall, the CSP family exhibits a relatively conservative number of genes, with nearly all analyzed species containing only four members (Figure 1). However, the number of OBP genes varies considerably among species, ranging from 37 to 66. The species within the *melanogaster* group (range = 47–66 genes) have more genes than those in the *repleta*, *virilis*, and *obscura* groups (range = 37–54 genes). *D. takahashii*, from the *melanogaster* group, possesses the highest number of OBP genes (*n* = 66), while *D. arizonae*, from the *repleta* group, has the fewest OBP genes (*n* = 37).

### 3.2. Phylogenetic Analysis and Gene Naming

**Figure 2:**
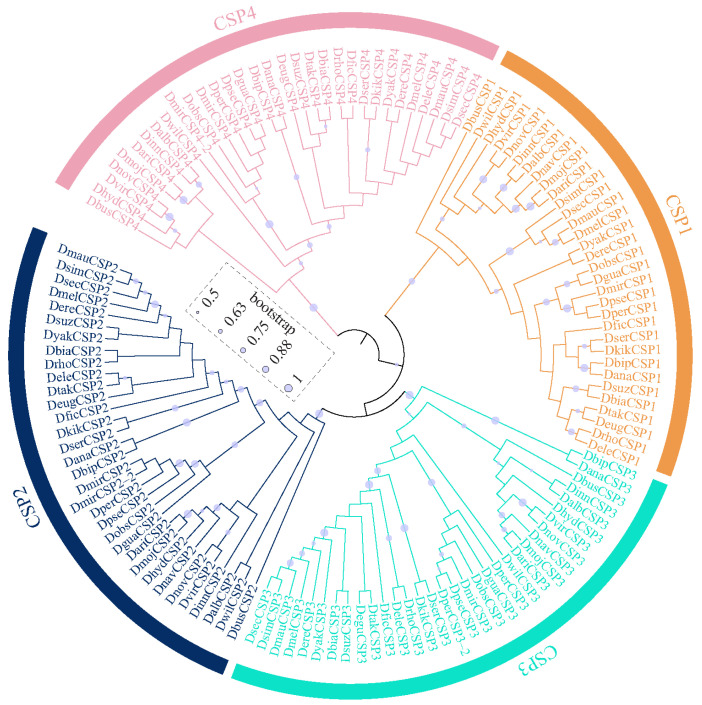
The maximum-likelihood phylogenetic tree of the CSP family in 32 *Drosophila* species.

**Figure 3:**
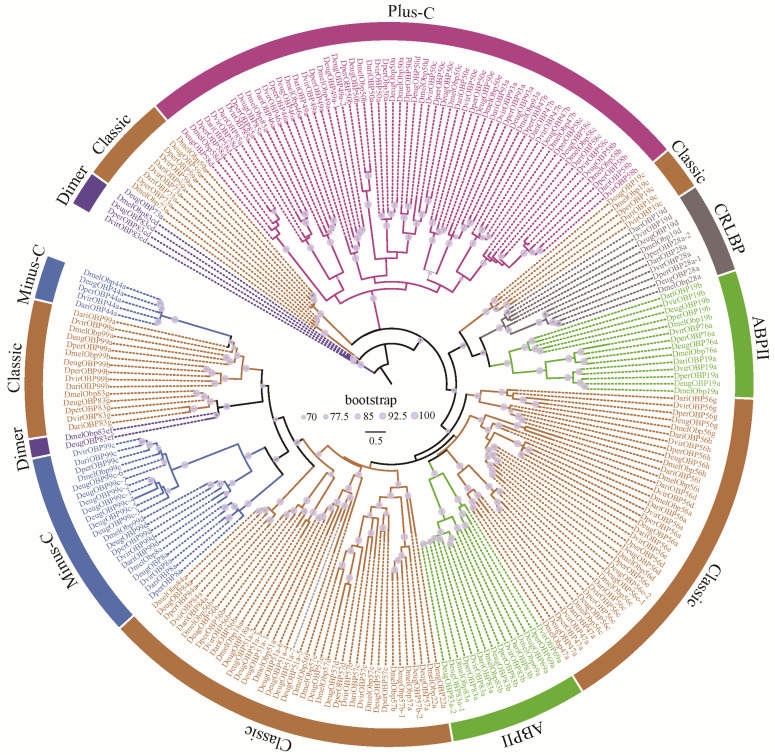
The ML phylogenetic tree of the OBP family in five representative *Drosophila* species. *Drosophila* OBPs were classified into 6 subfamilies, including Classic, Minus-C, ABPII, CRLBP, Dimer, and Plus-C [20].

The OBPs and CSPs of *D. melanogaster* have been systematically named [36,37], which serves as a reference for naming them in other species. All the CSPs were found to be grouped 4 clusters, corresponding to CSP1, CSP2, CSP3, and CSP4 in *D. melanogaster* (Figure 2). Similarly, *Drosophila* OBPs were classified into 6 subfamilies, including Classic, Minus-C, ABPII, CRLBP, Dimer, and Plus-C (Figure 3). Based on orthologous evolutionary relationships within the phylogenetic tree and nomenclature in the *D. melanogaster* [36,37], the identified CSP and OBP genes were named (Tables S2 and S3).

### 3.3. Gene Gain and Loss of OBP Family

To understand the evolutionary mode, the gene gain and loss of the OBP family were analyzed. Two major gene expansion events occurred at early nodes during the speciation of the *melanogaster* group, yielding two and seven new gene duplicates, respectively (Figure 1). Furthermore, another major expansion occurred at the ancestral node preceding the divergence of the four species, *D. eugracilis*, *D. biarmipes*, *D. suzukii*, and *D. takahashii*, yielding an additional nine novel gene duplicates. These multiple rounds of gene expansions have resulted in the *melanogaster* group possessing a relatively higher number of OBP genes. Gene loss primarily occurs at the terminal nodes of the evolutionary lineages or in specific species. For example, four genes were lost at the speciation node of *D. mojavensis*, *D. arizonae*, and *D. navojoa*. Similarly, the speciation node between *D. virilis* and *D. novamexicana* exhibited the loss of four genes. *D. biarmipes* lost as many as nine genes during the course of evolution.

**Figure 4:**
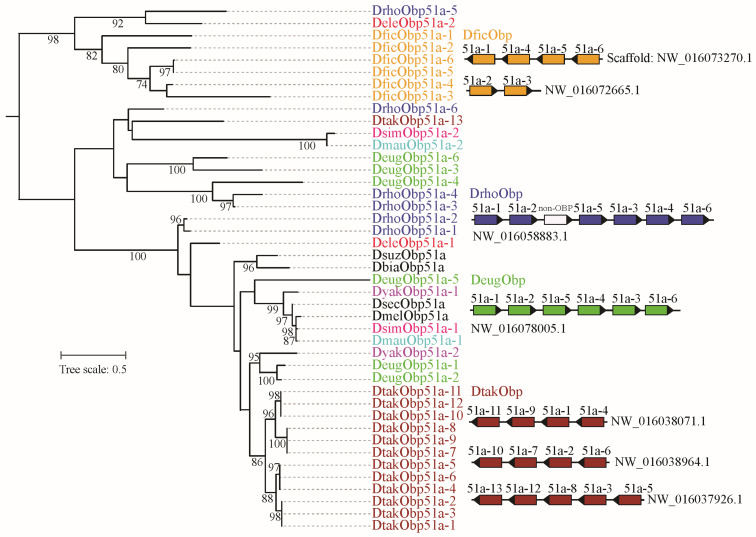
Phylogeny and chromosome distribution of orthologous gene group of *Obp51*. The ML tree was reconstructed by IQ-TRee v.1.6.12. The bootstrap values (>70%) were displayed. The *Obp51* was expanded in *D. ficusphila*, *D. rhopaloa*, *D. eugracilis*, and *D. takahashii*. The distribution of duplicated genes on the scaffold was presented. The non-OBP gene within the *DrhoObp51* gene cluster encodes other protein, namely non-OBP gene.

Based on gene gain and loss analysis, we found that the *Obp51a* gene underwent significant amplification in *D. takahashii*, resulting in a total of 13 copies (Table S2). We used all the *Obp51a* genes from the orthologous group to reconstruct the maximum likelihood tree (Figure 4). Except for *D. takahashii*, the *Obp51a* genes of the other three species were also duplicated. Phylogenetic analysis revealed that duplicated members tend to cluster together on the evolutionary tree within a species. Chromosome distribution analysis revealed that the *Obp51a* duplicates are tandemly distributed on a scaffold and share the same transcription direction in *D. eugracilis* and *D. rhopaloa*, respectively (Figure 4). The duplicated genes of *D. takahashii* and *D. ficusphila* are distributed across three and two scaffolds, respectively. However, the scaffold containing the duplicated *Obp51a* gene cluster is relatively short and may not yet have been assembled into a contiguous fragment. These results suggest that tandem duplication might be the primary reason for OBP gene expansion.

### 3.4. Genetic Divergence and Conservation of OBP and CSP Families

**Figure 5:**
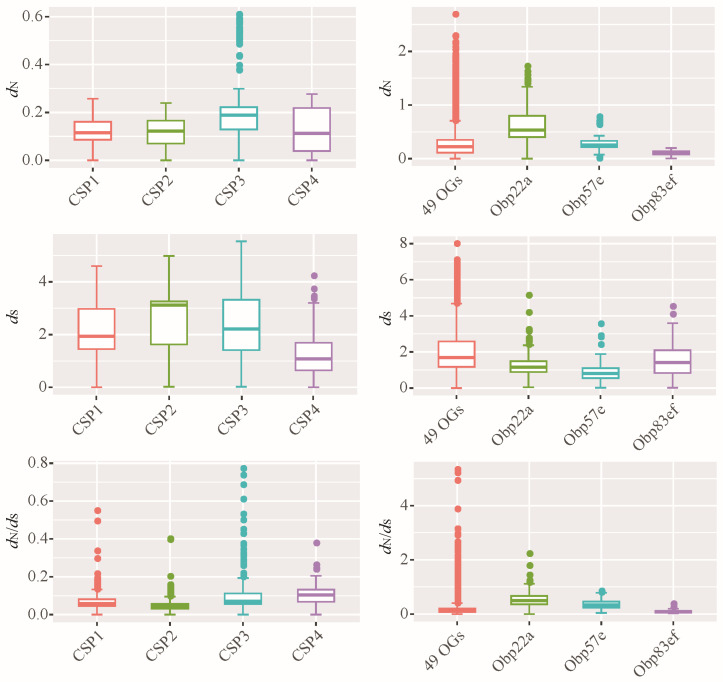
Evolutionary rates of orthologous groups of CSP and OBP families. *d*_N_: non-synonymous substitution rate; *d*_S_: synonymous substitution rate; the *d*_N_/*d*_S_ also known as ω value. Based on the ML phylogenetic tree, all the *Drosophila* CSPs were classified 4 OGs, including CSP1, CSP2, CSP3, and CSP4. Accordingly, all the OBP members were classified 52 OGs. For the OBP family, the evolutionary rates of 49 OGs were plotted together, namely 49 OGs. Additionally, three OGs each performed plotting, including *Obp22a*, *Obp57e* and *Obp83ef*.

Based on phylogenetic analysis, the CSP and OBP families were classified into 4 and 52 orthologous groups (OGs), respectively. In this study, genetic divergence was calculated for all the OGs, including non-synonymous substitution rates (*d*_N_), synonymous substitution rates (*d*_S_) and ω values (ω = *d*_N_/*d*_S_) with the YN00 program in the PAML [42]. For CSP genes, the non-synonymous substitution rates are relatively low, ranging from 0 to 0.61 (Figure 5). All CSPs contain the conserved four cysteine residues (Figure S1). Furthermore, the ω values of all CSP genes are less than one (Figure 5), indicating that CSP genes have undergone purifying selection during *Drosophila* evolution. Despite the overall evolutionary conservatism of CSP genes, relatively high ω values were observed in a few species. For example, the ω value for *DperCSP3* and *DmirCSP3* was 0.7725; the ω value for *DpseCSP3* and *DperCSP3* was 0.6864. This suggests that selective relaxation may have occurred in these specific species.

The average *d*_N_ of 52 orthologous gene sets of OBPs was reached to 0.28, which is significantly higher than the 0.14 observed for CSP genes (Wilcoxon rank test, *p* < 0.05). Similarly, the average ω of all OBP genes (0.18) was higher than that of CSP genes (0.08). These results suggest that *Drosophila* OBPs exhibit greater amino acid variation than CSPs. Additionally, some OBP genes, such as *Obp22a* (Figure 5), had ω values exceeding 1, suggesting the presence of positive selection.

### 3.5. Evidence of Positive Selection

All the orthologous groups of CSP and OBP gene families were used to detect signals of positive selection via CODEML in PAML. The results showed that three OBP OGs exhibited evidence of positive selection (Table 1). Relatively, *Obp22a* exhibited the highest number of sites potentially subject to positive selection, with 10 sites showing probabilities greater than 0.8. *Obp22a*, *Obp57e* and *Obp83ef* had 5, 1, and 1 positively selected sites, respectively, with probabilities exceeding 0.95. These results suggest that orthologous gene groups may have undergone functional diversification among species within the genus *Drosophila*.

### 3.6. The Effect of Positively Selected Sites on Ligand Binding Function

**Figure 6:**
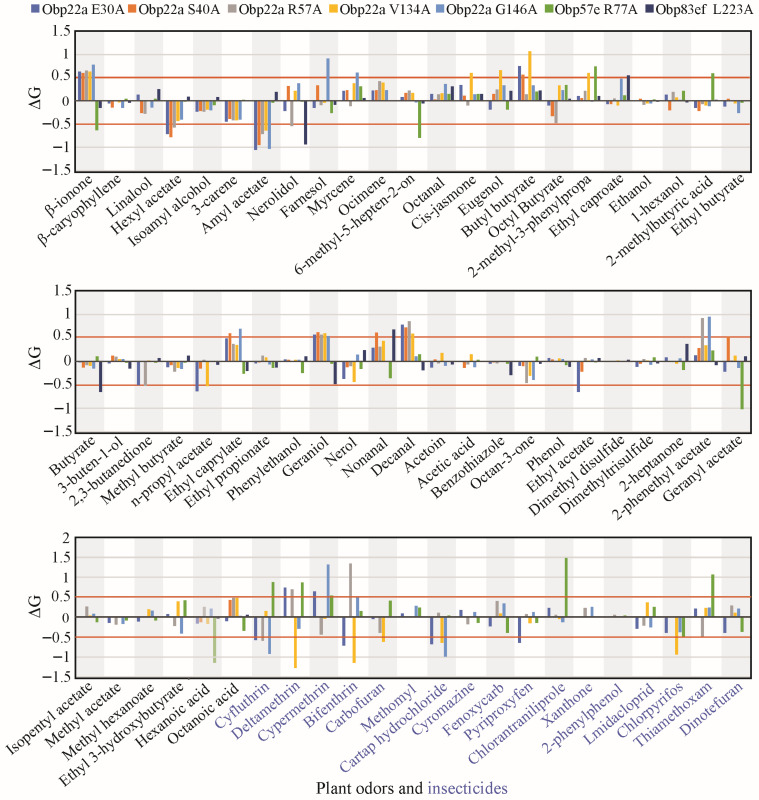
Statistics on computational alanine scanning and molecular docking at the positively selected sites of the three selected OBP genes. Only sites with a posterior probability ≥0.95 calculated using the BEB method were displayed, the other candidate sites potentially under positive selection were shown in Table S4. Plant odors were displayed in black font. Insecticides were displayed in blue font.

To investigate the impact of positive selection on odorant-binding function, this study employed homology modeling and molecular docking to simulate the differentiation of ligand-binding affinity among *Drosophila* species. DmelOBP22a, DmelOBP57e, and DmelOBP83ef proteins from *D. melanogaster* were used as template sequences to investigate the functional impact of sites under positive selection. The homology models were generated and evaluated using SAVES v6. Ramachandran plots revealed that the proportion of favorable regions in all three OBP models was 92.9% or higher (Figures S2–S4). CAS is commonly used to assess the degree of functional impact at specific amino acid sites [45]. Thus, we replaced all positively selected sites of DmelOBP22a, DmelOBP57e and DmelOBP83ef with alanine to analyze changes in free binding energy (ΔG). The analysis revealed that all of the examined sites exhibited an absolute ΔG value greater than 0.5 for at least one plant-derived odor or insecticide (Table S4, Figure 6). This suggests that the positively selected sites may play a significant role in the binding to specific ligands [44,45].

**Figure 7:**
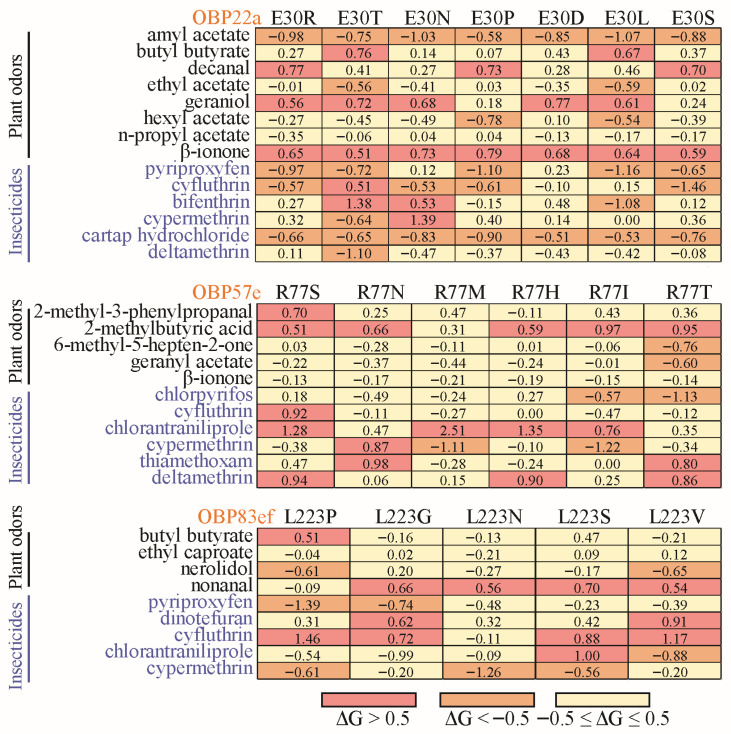
Heatmap of changes in free binding energy resulting from interspecies variation at the positive selection sites. The positively selected sites with a posterior probability ≥0.95 were used for site-directed mutation across *Drosophila* species (Table S5). For OBP22a, only position 30 was used for diagramming.

Under the influence of natural selection, positively selected sites often exhibit amino acid divergence among different species (Figures S5–S7). To gain deeper insight into the functional diversification, the positive selection sites were used for site-directed mutagenesis with corresponding amino acids from other *Drosophila* species (Table S5). For example, the glutamine (E) residue at position 30 of DmelOBP22a was replaced with one of seven variants (R, T, P D, L, S, and N) in other *Drosophila* species (Figure S5). Docking results showed that when E30 of DmelOBP22a was mutated to R, T, N, P, D, L, and S, the free binding energies to amyl acetate and cartap hydrochloride decreased, while the free binding energy to β-ionone increased (Table S5 and Figure 7). In addition to plant odor molecules, we also observed that variations at positively selected sites significantly influenced the binding capacity for certain insecticides (Table S5, Figure 7). Similarly, the positively selected sites in OBP57e and OBP83ef exhibited functional differentiation in response to certain plant odors and insecticides (Figure 7).

**Figure 8:**
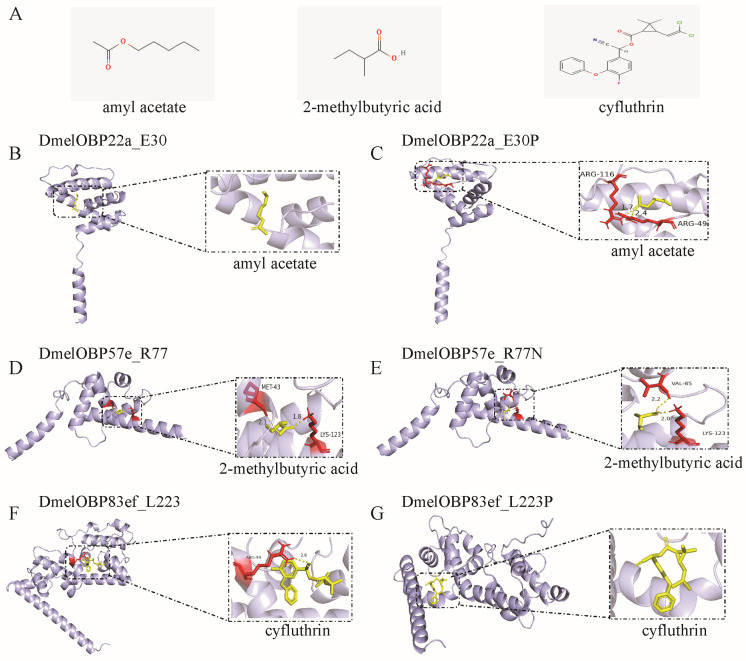
Docking of ligands into the binding pocket of the three OBPs undergoing positive selection and the interaction patterns. (**A**) Structure of the ligands. The amyl acetate and 2-methylbutyric acid are plant odors; cyfluthrin is insecticide. (**B**) Docking of amyl acetate into the binding pocket of DmelOBP22a. The site 30 of DmelOBP22a is Glutamate (E) (Figure S5). (**C**) Docking of amyl acetate into the binding pocket of DmelOBP22a after the position E30 is mutated to Proline. (**D**) Docking of 2-methylbutyric acid into the binding pocket of DmelOBP57e. (**E**) Docking of 2-methylbutyric acid into the binding pocket of DmelOBP57e after the position 77 (Arginine, R) is mutated to Asparagine (N) (Figure S6). (**F**) Docking of cyfluthrin into the binding pocket of DmelOBP83ef. (**G**) Docking of cyfluthrin into the binding pocket of DmelOBP83ef after the position 223 (Leucine, L) is mutated to Proline (P) (Figure S7).

Representative 3D and 2D diagrams of docking results were presented to demonstrate the effect of mutations at positively selected sites on ligand binding patterns (Figure 8 and Figure S8). Docking amyl acetate into the binding pocket of DmelOBP22a and DmelOBP22a_E30P revealed significant differences (Figure 8B,C). An E→P mutation occurs at position 30 of the DmelOBP22a protein was found to result in hydrogen bond interactions between amyl acetate and ar*g*inine 49 (Arg46) and ar*g*inine 116 (Arg116) (Figure 8C), thereby enhancing binding affinity and reducing the free binding energy (Figure 7). Conversely, the R77N mutation in DmelOBP57e and the L223P mutation in DmelOBP83ef resulted in increased hydrogen bond lengths or the absence of hydrogen bonds (Figure 8D–G). This resulted in increased free binding energies and reduced affinities for 2-methylbutyric acid and cyfluthrin in DmelOBP57e_R77N and DmelOBP83ef_L223P, respectively (Figure 7; Table S5). For instance, DmelOBP57e forms two hydrogen bonds with 2-methylbutyric acid, with bond lengths of 1.8 Å and 2.1 Å, respectively (Figure 8D). An R→N mutation occurs at position 77 of the DmelOBP57e results in variation at the two amino acid positions forming hydrogen bonds with the ligand and increases the bond lengths to 2.0 Å and 2.2 Å (Figure 8E). This results in an increase of 0.66 kcal/mol in free binding energy (Figure 7; Table S5). Site-directed mutations in OBPs can affect other intermolecular forces (Figure S8), such as alkyl and van der Waals forces, thereby influencing free binding energy and affinity. These results indicate that evolutionary variation at positively selected sites across different *Drosophila* species may play an important role in adapting to specific habitats and hosts.

### 3.7. Temporal-Spatial Expression Profiles in D. melanogaster

**Figure 9:**
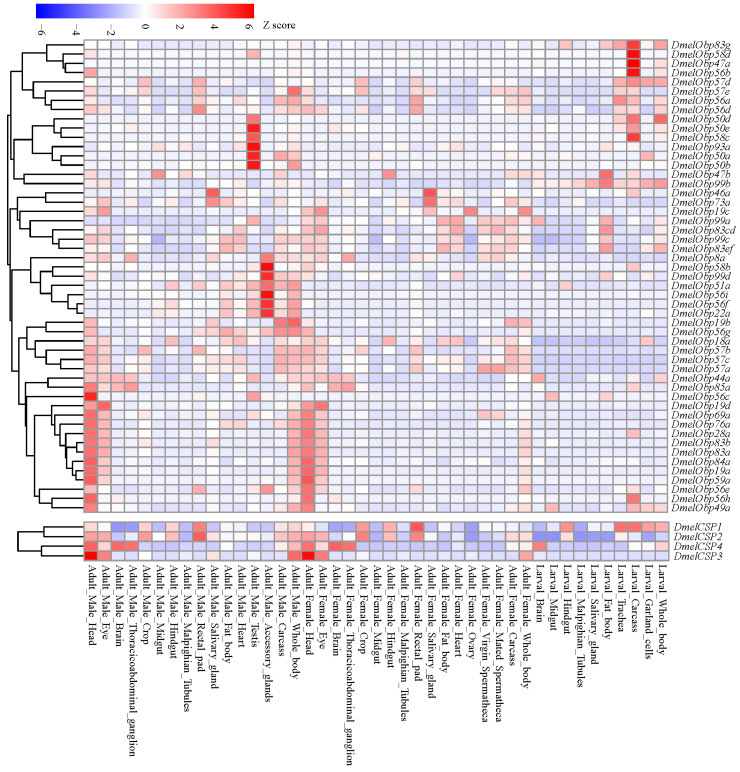
Temporal-spatial expression profiles of OBP and CSP genes in *D. melanogaster*. The expression signals (FPKM values) of all the OBP and CSP genes were downloaded from Flyatlas2 (https://motif.mvls.gla.ac.uk/FlyAtlas2/ (accessed on 5 November 2025)).

We analyzed expression profiles across different tissues and developmental stages of *D. melanogaster* to understand the potential functional diversity of OBPs and CSPs. Our results showed that members of the OBP and CSP gene families had similar expression profiles (Figure 9). Thirty-one of the 52 OBP genes and 4 CSP genes exhibited expression signals in the head of female and/or male adults. At the same time, many of those genes were also expressed in other tissues. For example, 26 OBP and 4 CSP genes were also expressed in the adult eyes. Conversely, some genes were not expressed in heads containing olfactory tissue, but were relatively abundant in the accessory glands or testis of adult males. In larvae, a certain number of OBP and CSP genes were also expressed in non-olfactory tissues, such as the carcass, trachea, and fat body. Analysis of expression patterns showed that OBP and CSP genes participate not only in the olfactory process but also in other important biological processes.

## 4. Discussion

OBPs and CSPs serve as transport carriers for chemical signals within the peripheral olfactory system. This study identified odorant-binding and chemosensory proteins in 32 *Drosophila* species, including *D. melanogaster*. A previous study identified OBP genes in 12 *Drosophila* species [36]. The number of OBPs identified in a few species differed slightly from those in this study, which may be attributed to the low-quality genome assemblies and fragmentation of OBP gene clusters across multiple scaffolds. The number of CSP genes is relatively conserved within the genus *Drosophila*, with approximately four genes per species. However, the number of OBP genes showed significant variation, ranging from 37 to 66 (Figure 1). Notably, the *melanogaster* group, comprising 17 species, experienced two rounds of gene expansion at ancestral nodes, acquiring two and seven novel OBP genes respectively (Figure 1). Furthermore, prior to the speciation of *D. eugracilis*, *D. biarmipes*, *D. suzukii*, and *D. takahashii*, a major expansion occurred, adding nine new genes. Conversely, specialist species such as *D. erecta* in the *melanogaster* group, which depend on *Pandanus candelabrum*, lost five OBP genes during speciation (Figure 1). Based on surveys of feeding and breeding resources, the *melanogaster* group has a broad dietary range, including fruit, flowers, other plant parts, and cactus [29]. This broad range of food sources may have led to the extensive expansion of OBP genes involved in host recognition processes.

CSPs within the genus *Drosophila* exhibited high conservation in both gene number and evolutionary rate (Figure 1, Figure 2 and Figure 5), suggesting that their functions may also be relatively conserved among *Drosophila* species. However, the OBP family exhibits relatively high gene numbers and sequence variation among *Drosophila* species (Figure 1, Figure 4 and Figure 5). Therefore, we performed a positive selection test on the 52 orthologous groups of the OBP family in *Drosophila*. The results indicate that only three orthologous groups (including *Obp22a*, *Obp57e*, and *Obp83ef*) showed significant positive selection, while the rest exhibited purifying selection across *Drosophila* species. For example, *OBP76a* and *OBP69a* play important roles in modulating social responses to the male-specific pheromone cVA [21,22]. Previous studies have suggested that cVA is one of the more common components of *Drosophila* aggregation pheromones, which is highly congruent with phylogeny [28]. Due to functional constraints or limitations, a large number of OBP genes in *Drosophila* still exhibit purifying selection. These results suggest that, during the adaptive radiation of *Drosophila* species, the gain and loss of OBP genes may play a dominant role rather than positive selection on orthologous genes leading to functional differentiation.

*OBP57e* was one of the positively selected genes that is co-expressed with *Obp57d* in the taste sensilla on the legs of *D. melanogaster* [24]. In *D. sechellia*, *Obp57e* is necessary for detecting hexanoic and octanoic acids, which are both involved in the oviposition site preference [24]. Replacing the *Obp57d/e* genomic fragment with that of *D. sechellia* resulted in a preference for octanoic acid at higher concentrations. Previous studies have shown that *OBP57e* has evolved rapidly in the *melanogaster* group [34,46]. Therefore, *OBP57e*, a gene subject to positive selection, may play a significant role in the adaptive evolution of host plants. Based on molecular docking, free binding energy changes at the positively selected site 77 exceeded 0.5 for four plant-derived molecules and six insecticides, reaching a significant level. For instance, substituting the arginine (R) at position 77 of *D. melanogaster* OBP57e with the corresponding serine (S) residue found in *D. biarmipes*, *D. elegans*, *D. suzukii*, and *D. takahashii* (Table S4) significantly increased the free binding energies to the plant-derived molecules, 2-methyl-3-phenylpropanal and 2-methylbutyric acid (Figure 7). This indicates a decrease in binding affinity for these compounds. Variations in the positively selected site 77 also significantly influence the binding of insecticides, such as cyfluthrin, chlorantraniliprole, and deltamethrin (Figure 7). Two OBP genes were overexpressed in *Myzus persicae* chlorpyrifos-resistant strains, RNAi knockdown of the two genes increased aphid susceptibility to chlorpyrifos [44]. Therefore, OBPs may competitively bind to toxic molecules in sensilla and remove toxic ligands, such as insecticides [6,7,44,47]. These findings indicate that, due to habitat variations among different *Drosophila* species, the types and doses of insecticides encountered may differ substantially. Changes in binding affinity resulting from variation at the positively selected sites may play a crucial role in maintaining olfactory sensitivity and conferring resistance to insecticides unique to particular habitats [48,49].

The ecological functions of the positively selected genes *Obp22a* and *Obp83ef* remain poorly understood. In *D. melanogaster*, *Obp22a* exhibited an olfactory response to 1-hexanol in females and to 2-heptanone in males [15]. *Obp83ef* is upregulated in the antennae of *D. sechellia* compared to *D. melanogaster*; however, its binding ligands remain unidentified [50,51]. However, neither of the two genes showed significant expression signals in the head tissue of *D. melanogaster* (Figure 9). This may be due to their lower expression in the antennae of *D. melanogaster*; despite the head containing antennae, this will further mask the expression levels, resulting in *Obp22a* and *Obp83ef* showing almost no expression throughout the entire head tissue. In this study, we found that the positive selection site 30 in the OBP22a significantly influenced changes in binding energy for eight plant-derived molecules and six insecticides (Figure 7, Table S5). Positive selection site 223 in OBP83ef significantly influenced the binding energies of four plant-derived molecules and five insecticides. These findings suggest that variations in specific sites of orthologous genes across *Drosophila* species may be crucial for adapting to different host plants through changes in the binding capacity for plant odor molecules. The geographical distribution and habitat differentiation among *Drosophila* species varies considerably, with widespread species being more readily exposed to insecticides [31]. The three positively selected genes exhibited significant divergence in binding affinity to several insecticides across different species (Table S5), suggesting that they may play a crucial role in adapting to insecticides to maintain olfactory sensitivity [9,48,49].

Analysis of expression patterns indicated that 31 OBP and 4 CSP genes exhibited relatively high expressions in the adult head of *D. melanogaster* (Figure 9), where the olfactory organs are distributed. Another study detected the expressions of 38 OBP genes in RNA samples isolated from the third antennal segment [50]. These findings further support the primary role of these genes in olfaction. However, not all OBPs and CSPs are restricted to chemosensory organs, suggesting that they may participate in other physiological functions. For instance, *Drosophila Obp56g* exhibits high expression in the male reproductive tract, which is involved in mating plug formation, ejaculate retention, and sperm storage [8]. In this study, *Obp56g* was expressed in the reproductive accessory gland (Figure 8). Furthermore, *Obp56g* underwent significant duplication in *D. guanche*, *D. obscura*, and *D. willistoni*, comprising 4, 5, and 5 members, respectively (Table S2). The positively selected gene *Obp22a* was also highly expressed in the reproductive accessory gland (Figure 8). In *Spodoptera frugiperda*, *OBP31* was found to be highly expressed in the male reproductive organs [52]. Following *OBP31* knockout, mating duration increased and offspring hatching rates decreased compared to wild-type individuals. Since reproductive systems often evolve rapidly, the association of the duplication of *Obp56g* and the positive selection of *Obp22a* with species and reproductive divergence requires further investigation. Additionally, there is direct evidence that OBP genes are associated with other traits, including gustatory responses to aversive (“bitter”) tastants [14] and lifespan [12]. For example, *Obp57d* and *Obp57e* are expressed in gustatory sensilla on the legs and are necessary for detecting hexanoic and octanoic acids, which participate in oviposition site selection [30]. CSPs expressed in non-olfactory tissues have been postulated that they are involved in carbon dioxide detection, larval development and leg regeneration [13]. Therefore, their widespread expression suggests that the OBP and CSP families are involved not only in olfactory processes but also in a variety of other physiological processes [48,53].

## 5. Conclusions

This study identified 1587 OBP and 130 CSP genes within 32 *Drosophila* species. Analysis expression profile indicated that the OBP and CSP genes are expressed not only in olfactory tissues, but also in other tissues, such as the eye, trachea, and fat body. This suggests they have diverse functions. The CSP family exhibits relatively conserved gene numbers across species, typically consisting of four members. All CSPs have four cysteine residues, and orthologous genes sequences are highly conserved, with an average non-synonymous substitution rate of only 0.14. However, the number of OBP genes varies considerably across species, ranging from 37 to 66. Gene gain and loss analyses revealed two major gene expansion events on ancestral branches of the diet-diverse *melanogaster* group, resulting in a greater number of OBP genes in this lineage. Furthermore, three orthologous gene groups (*Obp22a*, *Obp57e*, and *Obp83ef*) experienced positive selections during evolution. Molecular docking confirmed that variation in the positively selected sites across different *Drosophila* species causes significant changes in free binding energy with plant odor molecules and insecticides. These results suggest that the expansion of the OBP family and adaptive variation in orthologous genes play crucial roles in dietary shifts and adaptation to toxic substances within the genus *Drosophila*. Our research provides theoretical insights into the OBP family and identifies candidate genes for pest control.

## Figures and Tables

**Table 1:** Positive selection evidence for the OBP genes from the *Drosophila* genus.

Gene	M7 vs. M8		Sites with BEB pr > 0.80
	2*ΔlnL	LRT *p*-Value	
*Obp22a*	32.12	0.00000011	10V 0.820, 30E 0.987 *, 31E 0.913, 40S 0.994 **, 52E 0.839, 57R 0.992 **, 61L 0.892, 134V 0.977 *, 143I 0.876, 146G 0.996 **
*Obp57e*	16.79	0.00023	31S 0.816, 77R 0.962 *, 102T 0.812, 108S 0.930
*Obp83ef*	17.59	0.00015	202S 0.876, 223L 0.982 *

PAML analysis was under site model. LRT: likelihood ratio test. Amino acid residues with > 0.80 probability of being under positive selection are indicated. The number/letter indicates the position/amino acid of *D. melanogaster* in the sequence alignments. * probability > 0.95; ** probability > 0.99.

## Data Availability

The original contributions presented in this study are included in the article/Supplementary Material. Further inquiries can be directed to the corresponding authors.

## Supplementary Materials

The following supporting information can be downloaded at: https://www.mdpi.com/article/10.3390/biom16020330/s1, Figure S1: Multiple sequence alignment and conserved cysteine residues of CSPs in *Drosophila*. Figure S2: Ramachandran plot of quality evaluation of the DmelOBP22a structure model. Figure S3: Ramachandran plot of quality evaluation of the DmelOBP57e structure model. Figure S4: Ramachandran plot of quality evaluation of the DmelOBP83ef structure model. Figure S5: Sequence alignment of the putative proteins of the *Obp22a* OGs. Figure S6: Sequence alignment of the putative proteins of the *Obp57e* OGs. Figure S7: Sequence alignment of the putative proteins of the *Obp83ef* OGs. Figure S8: 2D schematic diagram of the interaction patterns between ligands and the binding pocket of the three OBPs undergoing positive selection. (A) and (B) Docking amyl acetate into the binding pocket of DmelOBP22a_E30 and DmelOBP22a_E30P. (C) and (D) Docking 2-methylbutyric acid into the binding pocket of DmelOBP57e_R77 and DmelOBP57e_R77N. (E) and (F) Docking cyfluthrin into the binding pocket of DmelOBP83ef_L223 and DmelOBP83ef_L223P. The 2D schematic diagram corresponds to the docking results of the ligand and protein shown in Figure 8. Various interactions between ligands and proteins, such as hydrogen bonds and van der Waals forces, are displayed using different dash lines. Table S1: The genome assembly information of 32 *Drosophila* species and download path. Table S2: *Drosophila* OBP genes and their accession numbers. Table S3: *Drosophila* CSP genes and their accession numbers. Table S4: Computational alanine scanning for the positively selected sites of Obp22a, Obp57e and Obp83ef. Each of the positively selected sites was replaced with alanine individually, and CAS was used to detect the change in free binding energy (ΔG) before and after the replacement. Table S5: Changes in free binding energy resulting from species differentiation at positive selection sites of OBPs. Based on CAS calculations, the absolute values of free binding energy changes (ΔG) for some ligands exceed 0.5 kcal/mol (Table S4). These ligands were used to further examine the effect of interspecies variation at the positive selection sites on free binding energy. The positive selection sites with a posterior probability ≥ 0.95 were used for site-directed mutation across *Drosophila* species.
