# Supplementary material for: Adaptive Evolution of Odorant-Binding and Chemosensory Protein Gene Families in Genus Drosophila Fallén, 1823 (Diptera, Drosophilidae)
Source: Biomolecules. 2026 Feb 20;16(2):330. doi: 10.3390/biom16020330 (PMC12937694; doi:10.3390/biom16020330)
Supplement: Supplementary file 1 [file biomolecules-16-00330-s001.zip › Figure S4.pdf]

PROCHECK

Ramachandran Plot

saves

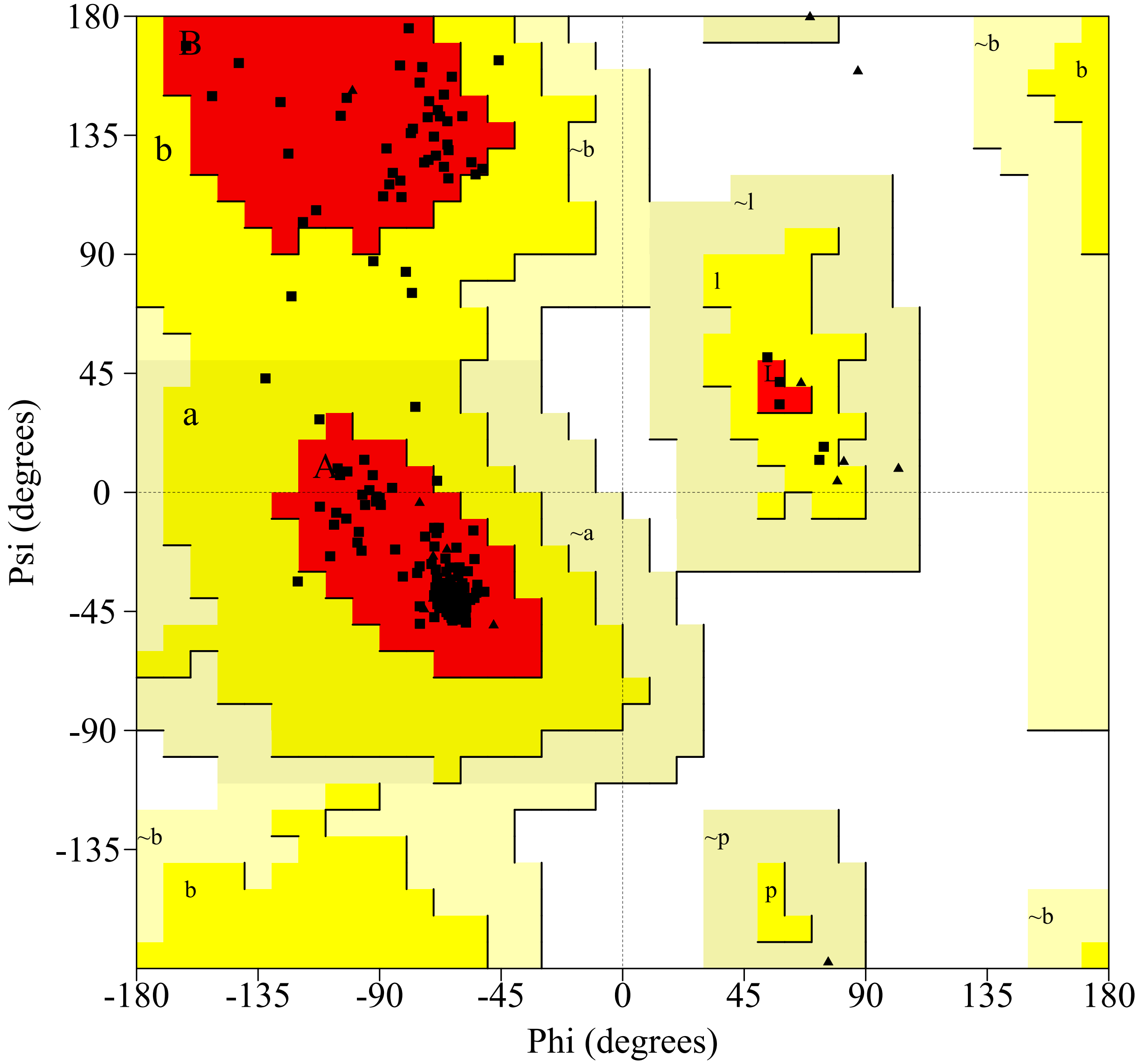

Plot statistics

Residues in most favoured regions [A,B,L]	207	94.1%
Residues in additional allowed regions [a,b,l,p]	13	5.9%
Residues in generously allowed regions [~a,~b,~l,~p]	0	0.0%
Residues in disallowed regions	0	0.0%
	----	------
Number of non-glycine and non-proline residues	220	100.0%
Number of end-residues (excl. Gly and Pro)	2	
Number of glycine residues (shown as triangles)	16	
Number of proline residues	7	

Total number of residues	245	

Based on an analysis of 118 structures of resolution of at least 2.0 Angstroms and R-factor no greater than 20%, a good quality model would be expected to have over 90% in the most favoured regions.
