# Supplementary material for: Adaptive Evolution of Odorant-Binding and Chemosensory Protein Gene Families in Genus Drosophila Fallén, 1823 (Diptera, Drosophilidae)
Source: Biomolecules. 2026 Feb 20;16(2):330. doi: 10.3390/biom16020330 (PMC12937694; doi:10.3390/biom16020330)
Supplement: Supplementary file 1 [file biomolecules-16-00330-s001.zip › Figure S5.pdf]

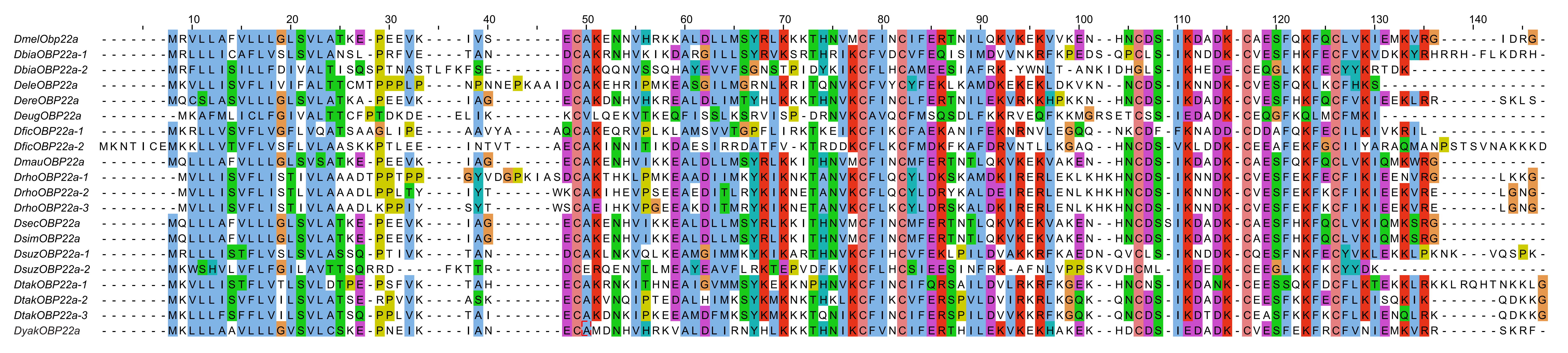
10
20
30
40
50
60
70
80
90
100
110
120
130
140
DmelObp22a
DbiaOBP22a-1
DbiaOBP22a-2
DeleOBP22a
DereOBP22a
DeugOBP22a
DficOBP22a-1
DficOBP22a-2
DmauOBP22a
DrhoOBP22a-1
DrhoOBP22a-2
DrhoOBP22a-3
DsecOBP22a
DsimOBP22a
DsuzOBP22a-1
DsuzOBP22a-2
DtakOBP22a-1
DtakOBP22a-2
DtakOBP22a-3
DyakOBP22a
