# Supplementary material for: Adaptive Evolution of Odorant-Binding and Chemosensory Protein Gene Families in Genus Drosophila Fallén, 1823 (Diptera, Drosophilidae)
Source: Biomolecules. 2026 Feb 20;16(2):330. doi: 10.3390/biom16020330 (PMC12937694; doi:10.3390/biom16020330)
Supplement: Supplementary file 1 [file biomolecules-16-00330-s001.zip › Figure S7.pdf]

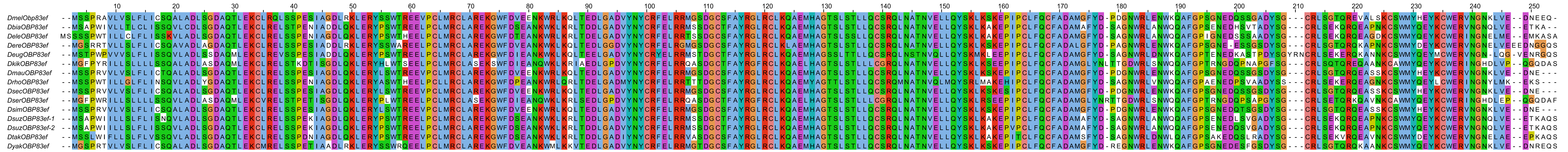
10 20 30 40 50 60 70 80 90 100 110 120 130 140 150 160 170 180 190 200 210 220 230 240 250
DmelObp83ef
DbiaOBP83ef
DeleOBP83ef
DereOBP83ef
DeugOBP83ef
DkikOBP83ef
DmauOBP83ef
DrhoOBP83ef
DsecOBP83ef
DserOBP83ef
DsimOBP83ef
DsuzOBP83ef-1
DsuzOBP83ef-2
DtakOBP83ef
DyakOBP83ef
