# Supplementary figures and images for: Adaptive Evolution of Odorant-Binding and Chemosensory Protein Gene Families in Genus Drosophila Fallén, 1823 (Diptera, Drosophilidae)
Source: Biomolecules. 2026 Feb 20;16(2):330. doi: 10.3390/biom16020330 (PMC12937694; doi:10.3390/biom16020330)

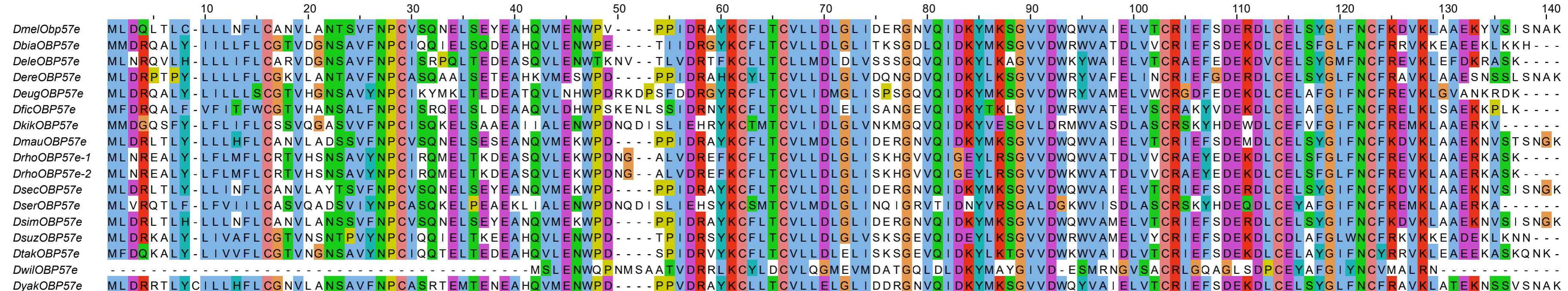

Supplement: Supplementary file 1 [file biomolecules-16-00330-s001.zip › Figure S6.pdf]

**A** DmelOBP22a\_E30

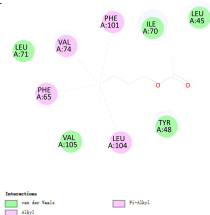

**B** DmelOBP22a\_E30P

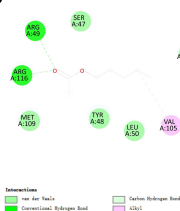

**C** DmelOBP57e\_R77

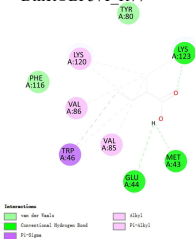

**D** DmelOBP57e\_R77N

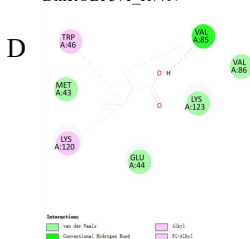

**E** DmelOBP83ef\_L223

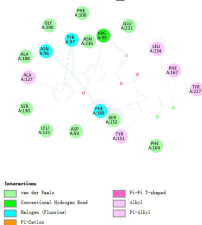

**F** DmelOBP83ef\_L223P

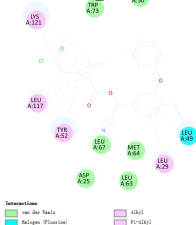

Supplement: Supplementary file 1 [file biomolecules-16-00330-s001.zip › Figure S8.pdf]
